# Amyloid-β and Phosphorylated Tau are the Key Biomarkers and Predictors of Alzheimer’s Disease

**DOI:** 10.14336/AD.2024.0286

**Published:** 2024-04-24

**Authors:** Jangampalli Adi Pradeepkiran, Javaria Baig, Md Ariful Islam, Sudhir Kshirsagar, P. Hemachandra Reddy

**Affiliations:** ^1^Internal Medicine Department, Texas Tech University Health Sciences Center, Lubbock, TX 79430, USA.; ^2^Pharmacology & Neuroscience Department, Texas Tech University Health Sciences Center, Lubbock, TX 79430, USA.; ^3^Neurology Department, Texas Tech University Health Sciences Center, Lubbock, TX 79430, USA.; ^4^Speech, Language and Hearing Sciences Departments, Texas Tech University Health Sciences Center, Lubbock, TX 79430, USA.; ^5^Public Health Department, Texas Tech University Health Sciences Center, Lubbock, TX 79430, USA.; ^6^Nutritional Sciences Department, College of Human Sciences, Texas Tech University, Lubbock, TX 79409, USA

**Keywords:** Alzheimer's Disease, Biomarker, Aβ, Tau, biofluids

## Abstract

Alzheimer’s disease (AD) is a age-related neurodegenerative disease and is a major public health concern both in Texas, US and Worldwide. This neurodegenerative disease is mainly characterized by amyloid-beta (Aβ) and phosphorylated Tau (p-Tau) accumulation in the brains of patients with AD and increasing evidence suggests that these are key biomarkers in AD. Both Aβ and p-tau can be detected through various imaging techniques (such as positron emission tomography, PET) and cerebrospinal fluid (CSF) analysis. The presence of these biomarkers in individuals, who are asymptomatic or have mild cognitive impairment can indicate an increased risk of developing AD in the future. Furthermore, the combination of Aβ and p-tau biomarkers is often used for more accurate diagnosis and prediction of AD progression. Along with AD being a neurodegenerative disease, it is associated with other chronic conditions such as cardiovascular disease, obesity, depression, and diabetes because studies have shown that these comorbid conditions make people more vulnerable to AD. In the first part of this review, we discuss that biofluid-based biomarkers such as Aβ, p-Tau in cerebrospinal fluid (CSF) and Aβ & p-Tau in plasma could be used as an alternative sensitive technique to diagnose AD. In the second part, we discuss the underlying molecular mechanisms of chronic conditions linked with AD and how they affect the patients in clinical care.

## Introduction

In Texas, Alzheimer's disease (AD) is a prevalent neurodegenerative disorder and a major public health issue with over 6.7 million, American, all ages included, living with AD in 2023, which is a major increase since 2020 with 400,000 Americans living with AD at the time. This ranks Texas as fourth in terms of AD cases nationwide behind California, Florida, and New York, in terms of deaths from AD, Texas ranks second [1-3].

Similar to the prevalence of AD in Texas, AD has also increased worldwide. In one study, the authors found that from 1990 to 2019, there was an increase in AD by 147.95% [4]. In terms of numbers, globally, there are 315 million and 32 million people with preclinical AD and AD dementia, respectively [5]. This makes AD one of the fastest growing health issues, making it critical biomarkers to be developed for AD.

Development of AD in patients is seen in several stages from no toxic amyloid β (Aβ) to accumulation of toxic amyloid beta with normal cognition. These stages occur for several years with no signs of AD and are known as preclinical stages of AD. Once the symptoms start appearing then the disease goes through mild, moderate, and severe stages with overlap between the stages, which make it difficult to place an AD patient in a specific spectrum [6]. Clinically, AD is characterized by memory loss, language deterioration, impaired visuospatial skills, poor judgment, and difference in attitude. These clinical symptoms occur 10-15 years after the onset of AD pathology: accumulation of Aβ 40 and 42 and Aβ protein in extracellular plaques in the brain and intra cellular phosphorylated tau (p-tau) [7].

Pathologically, AD is characterized by Aβ, in form of Aβ40 and Aβ42, and phosphorylated Tau-rich neurofibrillary tangles (NFT) in the brain. Accumulation of amyloid plaques is done via interaction between amyloid precursor proteins (APP) and two proteases: beta (β-) and gamma (γ-) secretases. Normally, APP is cut by alpha (α-) and γ- secretases, forming soluble APP, which does not form plaques. However, when APP interacts with β and γ-secretases, this results in increase in Aβ production, which forms the insoluble APP. Insoluble APP clump together to form amyloid plaques [8-10] Aβ and other factors such as phosphorylation/ dephosphorylation pathway dysregulation results in hyperphosphorylated tau (τ). Both accumulation of Aβ and p-tau can increase generation of ROS and induce mitochondrial dysfunction [11, 12].

In the AD brain, NFT and Paired helical filaments (PHF) are composed of hyperphosphorylated Tau (τ). Phosphorylated Tau is a microtubule-associated protein that regulates the organization of neuronal microtubules [13]. In its pathogenesis, AD is considered as genetic and multifactorial. For example, some of the AD cases show an autosomal dominant. In early onset familial AD cases, mutations in the genes encoding presenilin 1(PS1) and presenilin 2 (PS2) and AβPP have been characterized. The well-known risk factors for AD are ageing, genetics, health conditions and diseases, lifestyle factors, and a positive family history of dementia. Biomarker studies suggest that Aβ accumulation in the brain and Aβ leakage in peripheral blood can be monitored with an increased phosphorylation of tau and have been targets for biomarker development for two decades in AD human studies. However, in terms of biomarker development, the levels of Aβ peptide and tau in the blood for diagnosis of AD has not been established. Furthermore, detection of AD progression in plasma or serum using multiplex or ELISA is difficult due to high levels of assay-interfering factors [14]. The present review originally represents that AD biomarkers could be used as an alternative sensitive technique but due to technological progress, it is now possible to measure them in standard blood samples as well. The present review also covers that AD blood-based biomarkers reflect the key components of AD pathology.

## Biomarkers for Amyloid beta (Aβ) Pathology

The peptides of BACE (β-site APP cleaving enzyme) gene with 37-49 amino acids are the main component for generation of the amyloid plaques in the brains of AD people. The peptides derived from the APP are Aβ 40 and 42 peptides through cleavage by two proteases β- and γ-secretases, resulting in accumulation and release of amyloid plaques into the extracellular space [15, 16]. The γ-secretase cleavage site determines the length of the Aβ peptide, sizes from 37 to 49 residues length [17-20]. The amplest Aβ isoforms in CSF are Aβ1-38, Aβ1-40, and Aβ1-42 [21,22]; with the 1-40 and 1-42 length, isoforms were most extensively studied in AD. The length isoforms Aβ1-42/Aβ1-40 ratio (Aβ1-42/1-40) play an important role in diagnosis of AD, as emphasized by the neurodegeneration and AD biomarker development process [23,24]. In CSF, concentrations of Aβ1-40 remain unchanged and Aβ1-42 concentrations decrease in AD, this aggregation and deposition of Aβ 42 within the AD brains, helps to detect AD brain samples [25,26]. Therefore, looking at these two combinations Aβ 40 and 42 offers a more accurate AD biomarker developmental process in comparison to the overall Aβ production in human AD brain samples, and combats issues at Aβ1-42 concentrations in human CSF samples of AD [26].

However, within Aβ plaques in both familial Alzheimer’s disease (FAD) and late-onset sporadic AD postmortem brains, longer-length Aβ peptides, such as Aβ 43, have been observed more frequently than Aβ 40. In post-mortem analysis of human AD brains, there was a positive correlation between length of Aβ peptide and plaque load order (Aβ 43 > Aβ 42 > Aβ 40), and studies in FAD mouse models have revealed that Aβ43 is more neurotoxic and has a greater propensity to aggregate than Aβ1-42 [27]. In AD patients, CSF concentrations of Aβ1-43 were significantly reduced in FAD mutation carriers, mimicking the reduction in Aβ 42 seen in AD, and underlining a potential role of Aβ 43 in AD [28, 29].

The above referred Aβ formation and deposition in the C-terminus of Aβ are a well-known phenomenon. The amyloidogenic processing of APP is carried out by the sequential reaction of membrane bound β- and γ-secretase cleavages. Where β-Secretase cleaves APP into the membrane bound C-terminal fragments β (CTFβ or C99) and N-terminal sAPPβ, and CTFβ is subsequently cleaved by γ-secretases into the extracellular Aβ and APP within intracellular domain (AICD). However, there are comparable differences at the N-terminus and C-terminus of the Aβ peptide with α-helix conformation between amino acid residues [30, 31]. Earlier investigations have revealed that only a small proportion of the Aβ end at amino acids 40 and 42 within CSF blood and parenchymal vessels of neocortical regions., respectively [32,33]. The truncated species, which include pyroglutamate modified Aβ (AβpE), is one of the dominant forms of Aβ in the brain hippocampal region and cortex of AD patients [34, 35]. By comparing it to full-length Aβ, AβpE that has been truncated and modified at the third amino acid of Aβ (Aβp_E3_) has shown to be significantly increased in rate of aggregation in AD samples [34; 36]. Additionally, AβpE is the only identified form of Aβ that is solely found within plaques and is formed by glutamine or glutamate residues. AβpE is one of the interesting Aβ isoforms that has been shown to be dominant, present in the hippocampus and cortex region of AD patients and considered as promising Aβ biomarker.

## Aβ Isoforms

Aβ peptides are the main components of amyloid plaques. The biochemical and molecular properties of Aβ help us to better understand AD pathogenesis at the molecular level. Aβ peptides exist in different isoforms including Aβ monomers, oligomers, and regular fibrils. The Aβ peptides share a common structural motif and aggregation pathway, providing a powerful conceptual framework for understanding the pathogenic mechanism and disease-specific factors of AD. Monomeric form of Aβ exists in both α-helical and β-pleated sheet, and is amphipathic in nature, exhibits hydrophobicity at the N-terminal region, and hydrophobicity at the C-terminus region [37, 38]. These monomeric isoforms can later aggregate to form soluble oligomers, which are heterogenous in size and can spread throughout the brain. There are insoluble fibrils, which can further aggregate to form Aβ plaques [39]. All these aggregated forms of Aβ forms are known to be neurotoxic [40, 41, and 42]. Fibril formation is now widely considered to occur by nucleation-dependent polymerization [43]. However, Aβ42 is much more prone to aggregation and plaque formation in brain, it showed five-fold lower minimum concentration to aggregate into fibrils than Aβ40, and Aβ42 is much more abundant in plaques than Aβ1-40 showed in brain and CSF sample of human AD samples.

## Aβ Isoforms in Cerebrospinal Fluid

Via proteolytic processing by β- and γ-secretases, Aβ is formed as a cleavage product of APP. In this process. Aβ isoforms of 36-43 amino acid residues in length are generated when γ-secretase cleaves within the transmembrane region of APP. In the CSF, there are three major forms of Aβ detected: Aβ38, Aβ40, and Aβ42 along with other minor abundance forms [44, 45]. Although Aβ40 is produced at higher levels, Aβ42 is more hydrophobic and prone to form aggregates. Importantly, Aβ42 is more neurotoxic and seems to be of particular importance for plaque formation, while Aβ40 appears to be the most abundant peptide deposited in cerebral vessels [46]. Furthermore, it has been shown that both the relative concentration of Aβ42 and total Aβ concentration are critical factors in the rate of Aβ production in AD amyloidogenesis.

## Aβ Oligomers

While the original amyloid cascade hypothesis was introduced and developed, the detection of amyloid-β oligomers (AβOs) in human brain parenchyma and vasculature was first reported. Currently, AβOs are widely regarded as the most toxic and pathogenic form of Aβ [47-49]. In humans and animal models, AβOs show an AD-dependent presence, and their buildup occurs before the development of plaques, evidenced by both immunochemistry and immunohistochemistry. Furthermore, an ultrasensitive assay, known as the BioBarcode, which is capable of attomolar measurements, showed median levels of AβOs in CSF from AD patients to be 30-fold higher than from non-demented individuals. Many reports strongly suggest that AβOs are both necessary and sufficient for dementia.

## BACE1

Beta-secretase 1 (BACE1), also known as beta-site APP cleaving enzyme 1, membrane-associated aspartic protease 2, memapsin-2, aspartyl protease 2, and ASP2, is an enzyme encoded by BACE1 gene in humans. Furthermore, BACE1 is an aspartic acid protease that plays an important role in the myelin sheaths formation in peripheral nerve cells [50]. Additionally, BACE1 also plays as important role in the generation of Aβ peptides in the neurons as a major β-secretase.

## Amyloid Beta Precursor Protein Processing

**Figure 1.**
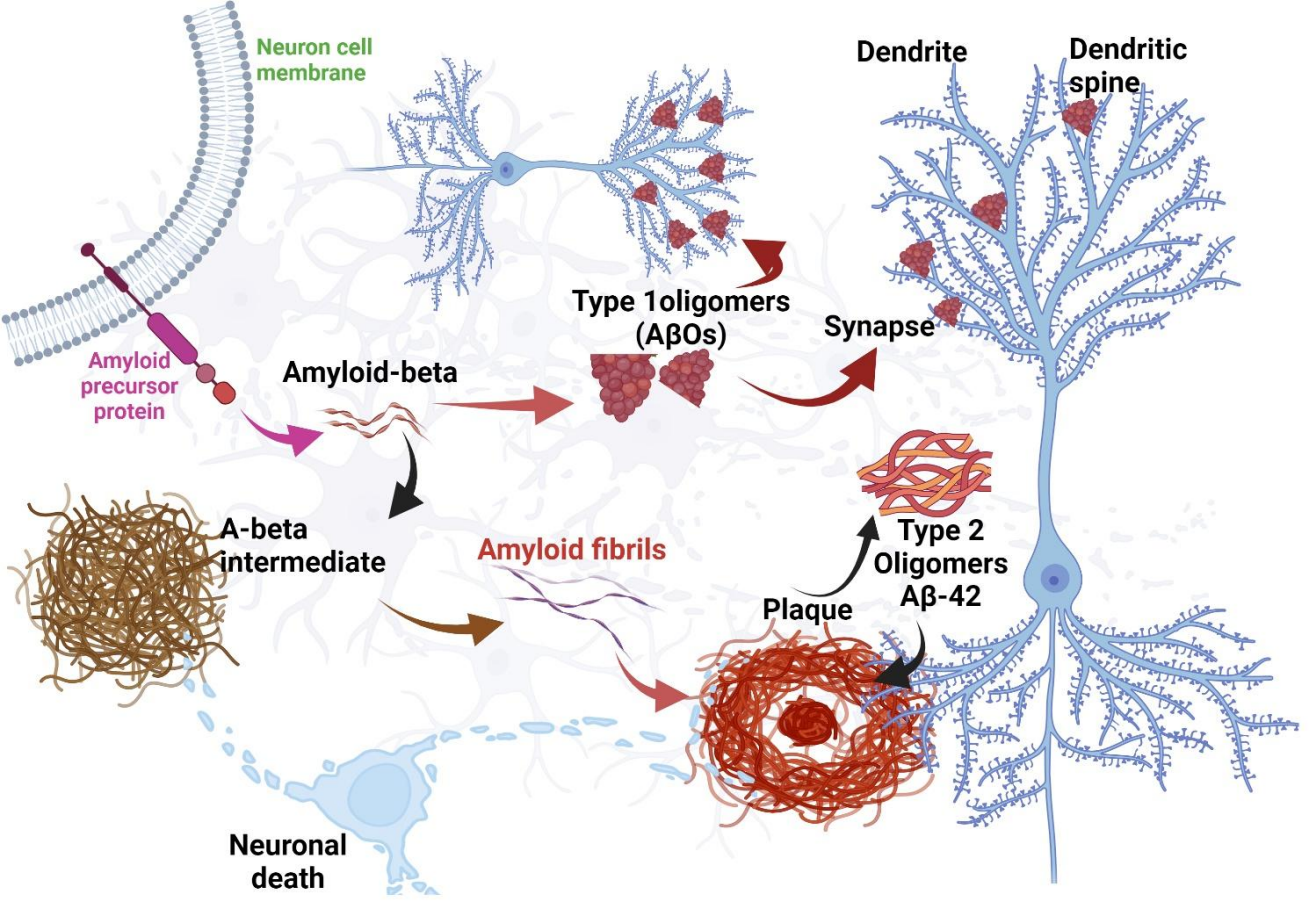
**Pathological Aβ substrates in Alzheimer disease (AD)**. This is a schematic diagram summarizing the amyloid hypothesis most widely examined biomarkers that reflect different pathologies in AD Amyloid-beta oligomers type 1 (AβOs) and type 2(Aβ42), Aβ intermediate and amyloid fibrils and with amyloid plaques.

During the proteolytic processing of APP, β-secretase cleaves APP at the amino terminus of Aβ, producing a secreted form of APP (sAPPβ) and membrane-bound C99. Then C99 is cleaved by γ-secretase to generate Aβ and intracellular carboxy-terminal fragment (CTF) γ. During the process, C89 and truncated amyloid regions could be produced via cleavage of APP by β-secretase within the Aβ region, but most APP undergo a non-amyloidogenic cleavage process [51]. To produce a secreted form of APP (sAPPα) and membrane-bound C83, α-secretase cleaves APP within the Aβ domain. Then γ-secretase cleaves C83 further, producing extracellular fragment p3 and intracellular CTFγ. BACE1 is the β-secretase in vivo, and the components of the γ-secretase complex are presenilin (PS), nicastrin (NCT), presenilin enhance 2 (Pen-2) and anterior pharynx-defective 1 (Aph-1). The activity of α-secretase is associated with several members of the ADAM (a distintergrin and metalloproteinase) family: ADAM9, ADAM10 and tumor necrosis factor-α convertase (also known as ADAM17), though other proteases may also contribute [52]. Through APP processing, soluble APPs, such as sAPPα and sAPPβ, are produced. In the non-amyloidogenic process, α-secretase cleaves APP to release soluble APPα and C83 fragments. Then C83 is subsequently cleaved by γ-secretase complex. This sequential processing fails to produce Aβ. In contrast, the amyloidogenic pathway is initiated by β-site APP cleaving enzyme (BACE1), which leads to the release of soluble APPβ and the C99 fragment. The C99 fragment is subsequently cleaved into Aβ and APP intracellular domain (AICD) by the γ-secretase complex. BACE1 protein and activity levels are elevated in the brains and platelets of AD patients [53,54]. Therefore, soluble APPs could be a potential biomarker that reflects specific features of AD-related pathophysiology (Fig. 1).

## Structure and Function of Tau

The structure of the tau protein includes multiple isoforms, each of which has a distinct function in the human brain with an alternative splicing [54]. Different combinations of exons characterize each of the six isoforms, referred to as tau A to tau G. The tau protein with ID: P10636 has 758 base pairs; this tau protein promotes and regulates the assembly of microtubules and the stability of the assembly by promoting the apical-basal polarity of neurons, which involves the propagation of signals in a dendrite to a particular direction (and axon). The microtubules are formed from the polymerization of α- and β-tubulin and are arranged in a polarized manner with plus and minus ends. This protein links kinesin superfamily proteins (KIFs) and cytoplasmic dynein, the motor proteins play key role by promoting the transport of mitochondria through the acquired ATP hydrolysis mechanism [55,56]. This binding may involve plasticity and cytoskeleton stability in the adult brain. Previous research had found binding domains on the C-terminal side of the microtubule protein.

## Interaction Between Tau and Microtubules

The interaction between tau and microtubules is pathogenic in AD and several other neurodegenerative diseases [57,58]. The microtubule is a cytoskeletal component in the cytoplasm and is comprised of α and β tubulins. Tau is a heterodimer, ratio of 1:2 that can grow in a dimer, where α and β tubulins are highly dynamic and are tightly bound to microtubules [59,60]. Tau uses a conserved of microtubule polymerization to regulate axonal stability and cell morphology. These microtubules have important functional properties in the cell, where it maintains cell structure and regulates intracellular transport of cargos. In the neuron, tau promotes the mobility of secretory vesicles, organelles, polarity, and the organization of macromolecules such as kinesin and dynein [58, 61]. The impaired interaction of tau with microtubules has been found in several neurodegenerative diseases, including AD [62, 63]. In problematic binding of Tau-mediated tubulin heterodimers in microtubule assembly lowering the critical concentration of tubulin polymerization. Where the binding vicinity of Tau with polymerized and un-polymerized tubulin. The variations of single nucleotide polymorphisms (SNP) lead to unstable microtubule structures, eventually resulting in neuronal death or neurological disorders associated with dementias [64].

## Tau Aggregation

The six isoforms of Tau contain 37 to 47kDa residues, characterized by conserved repeat regions and 5 to 6 base pairs [65, 66]. These isoforms residues are mostly towards the terminal ends of the N-terminal or C-terminal, where long isoforms repeat. These repeats may play a key role in microtubule stabilization [67]. The (3R) or (4R) tubulin binding domains of amino acids are in the C-terminal and in the N-terminal with no inserts of the molecule [68]. Researchers have hypothesized that tau domains hold the rich residues of prolines, which are amino acids that bind to microtubules [69]. The six-tau isoforms are located at four different domains: two MAP2 and two MAP1 [70]. The first MAP2 domain is located from 561 to 591, and the second, from 592 to 622. The first MAP1 domain is located from 623 to 653, and second from 654 to 685. These four domains play an important role in the stabilization of the microtubule assembly [71].

All tau isoforms contain a conserved tubulin-binding domain, which is located within the cl02863 family of all vertebrate MAP2, MAP4, and tau domains. The MAP4 domain is found in almost all vertebrate animal tissues, but it is absent in neurons, in contrast to the MAP2 and tau domains, that are mainly found in neurons [71]. Research has indicated that the conserved motif KXGS is present in all tau isoforms [72]. This motif is a key participant in the cAMP-dependent kinase that is located within cl02863. The KXGS motif contains a domain that binds tau at the C-terminal end of the MAP2 domain to form an unstable PHF that has been found in many dementias [73].

## Significance of MAP2 in Neuronal Growth in Relation to Tau and Synaptic Plasticity

**Figure 2.**
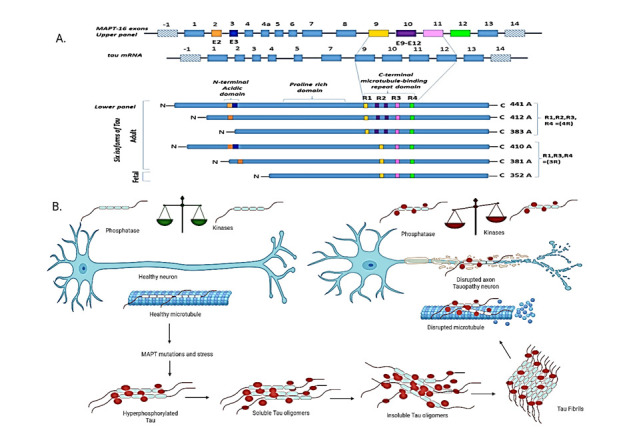
**Pathological Tau is regulated by phosphorylation via the concerted action of kinases and phosphatases**. (**A**). The tau contains 16 exons, with exon -1, a part of the promoter (upper panel). The tau primary transcript contains 13 exons. Exons -1 and 14 are transcribed but not translated. Exons 1, 4, 5, 7, 9, 11, 12, and 13 are constitutive, and exons 2, 3, and 10 are alternatively spliced and give rise to six different mRNAs (five adult and one fetal), translated in six different CNS tau isoforms (lower panel). In the adult human brain, the MAPT gene has six predominant isoforms of tau. These isoforms differ in the absence or presence of one or two N-terminal inserts of 29 amino acids encoded by exon E2 (orange box) and exon E3 (blue box). The MAPT gene combines with three (R1, R3, and R4) or four (R1, R2, R3, and R4) C-terminal repeat-regions (black boxes). The additional tau microtubule-binding domain is encoded by exon 10 (purple box, lower panel), which is found in only three isoforms (441, 412, and 383). Four isoforms (441, 412, 410, and 381) have been found in the adult human brain, and isoform 352, in the fetal human brain. Three functional domains have also been found: acidic, proline-rich, and repeat. The acidic domain is located at the N-terminal, followed by the proline domain located between the repeat domain at C-terminal region, and the acidic and repeat domains in the MAPT region. (**B**). Unbalance between phosphatases and kinases leads to microtubules function normal in healthy neuron. Imbalanced phosphatases and kinases leads to disruption of microtubules, hyperphosphorylated Tau, misfolding Tau drives oligomerization and aggregation leads to Tau fibrils leads neuronal death in AD.

The MAP2 family is a unique class of structural proteins that control microtubule dynamics in neurons during the development of dendrites and axons. MAP2 was originally associated with microtubule polymerization [74]. MAP2 is an important regulatory element of tau that triggers extracellular signals and helps in the formation and stabilization of cytoskeletons. MAP2 results from the splicing of a single MAP gene. Based on molecular weight, MAP2s are divided into four types: MAP2a, MAP2b, MAP2c, and MAP2d. Among the four, MAP2c is expressed mainly in rats and is associated with maximal dendritic outgrowth and synaptogenesis [75]. MAP2c binds with microtubules and is involved in maintaining and at times reducing the structural stability of the cytoskeleton *in vitro.* The MAP2c ultimately results in microtubule lengthening. MAP2c at high levels into maturity, signifying that MAP2c involves functional aspects accompanying dendritic growth and synaptogenesis. MAP2 is also involved in stability through controlling the tubulin polymerization dynamics. The cytoplasmic Ser/Thr-dependent phosphokinases significantly influence the reduction or promotion of MAP2 binding to microtubules [76]. The MAP2 binding affinity to actin filaments through crosslinking activity, which is regulated by the phosphorylation activity, has been reported, and the mechanism and sites involved in phosphorylation regulation of the actin association are not known. It is clearly shown in many studies that hyperphosphorylation and/or specific mutations can promote aggregation of the dissociated tau into PHF followed by NFT in many neurodegenerative diseases including AD (Fig. 2).

## Dynamics of Tau Binding Sites

Tau dynamics significantly contributes to the progression of AD by promoting pathological neural degradation. This degradation involves tau dissociating from microtubules and creating insoluble PHFs. Tau plays a conspicuous role in regulating microtubular dynamics and axonal development. Tau is comprised of nearly 80% serine, threonine, and tyrosine. Sites of tau phosphorylation are regulated by different kinases; the specific residues are involved in unstable structure of microtubules. Different combinations of tau residues (Thr212, Thr231, and Ser262) result in neurodegeneration or tau phosphorylation [77,78]. The six isoforms of tau and their combinations are expressed differentially and have distinct functions. Thr212 is the main phosphorylation site, and combinations of Thr231 and Ser262 are very important in binding stable microtubules [79].

It is difficult to determine which amino acid is responsible for the hyper-phosphorylation of tau, but this hyperphosphorylation is associated with certain phosphokinases [80]. The involvement of tau in phosphorylation takes place through the activation of messengers that target Ser, Thr, and Tyr residues. The proline-directed kinases target Ser and Thr, and other kinases to regulate tau stabilization of unstable microtubules [69,77].

## Tau Protein Structure

The structure of tau is hard to characterize due to its untranslated regions, alternative splicing including immature RNA sequence. The tau protein encompasses 441 amino acid residues in a single gene located on chromosome 17q21 with 16 exons. Of the 16 exons in humans, 13 have been successfully transcribed to form the mature sequences 4A, 6, and 8 [81]. The -1 and the 14 exons have been transcribed but not translated to form mature mRNA, whereas the 1, 4, 5, 7, 9, 11, 12, and 13 exons have been transcribed and translated. Only the 2, 3, and 10 exons are responsible for the formation of six important tau isoforms in the brain neurons of adult humans [82] These six isoforms differ in three or four tubulin binding domains within the C-terminal region of microtubules where they are responsible for binding. The adult tau isoform includes 441 residues of tau protein [83]. The tau protein contains four constitutive parts: 1) The N-terminal region, from 1 to 200, 2) A proline-rich region, from 200 to 244, 3) a microtubule-binding region, from 244 to 369, and 4) the C-terminal region, from 367 to 441, which is considered the dominant structure of tau.

## Biochemistry of Tau

Biochemical studies have revealed that microtubule stabilization depends on the dimerization of tau, on the negatively charged N-terminal domain of one tau molecule interacting with the positively charged proline-rich residues of a neighboring tau molecule [84]. This N-terminal domain is considered a region where tau regulates phosphosites of microtubules. Thus, this domain is called a projection domain [85]. The expressions of tau isoforms in fetal and adult human brains are difficult to distinguish because R3 and R4, which are repeats, are expressed in fetal and in adult brains in equal ratios. In addition, tau mutations found in persons with dementia influence the expression of R3 and R4 in adult human brains [86]. The N-terminal of the tau protein is associated with the plasma membrane, which is the main source for many kinase substrates [87]. Recent studies have found that the microtubule-binding domain may be the site of tau phosphorylation through the inhibition of tau phosphatase activities [88].

## Phosphorylation and Hyperphosphorylation Sites of Tau

In the tau isoform, there are 79 to 80 Ser, Thr, and Tyr phosphorylation sites. At these sites are a variety of Ser and Thr phosphatase residues, all involved in phosphorylation, such as phosphatase protein1, 2A, 2B, and 2C [89]. In addition, nearly 30 to 32 Ser or Thr residues are involved in the inhibition of phosphatase 2A in brain neurons. Many kinases, like proline-directed protein, MAPs and cyclin-dependent kinases are involved in phosphorylation regulation in the brain [90]. There is a counterbalance between kinase and phosphatase proteins that play normal physiological state of the brain in an association of microtubules. Furthermore, Tau phosphorylation in brain cells has been found in human fetal brains [91].

A biological property of tau is its ability to affect the development and stability of microtubules. Tau is associated with an imbalance of kinases and phosphatases in microtubules, which leads to tau aggregation and the instability of microtubules, precursors to NFT [92]. In AD, irregular hyperphosphorylation of tau appears to precede its accumulation in infected neurons. Irregular hyperphosphorylation may also block the transport of nutrients to microtubules.

Phosphorylation occurs in the sites of kinases, such as protein kinase A (PKA), protein kinase C (PKC), calmodulin-dependent protein kinase (CaMK), and most of the phosphorylation regions encode at T153 to S253. CDKs are at sites S235, S202, and S404 [93]. Other examples of phosphorylation include Ser214, 324, 356, 409, and 416; Ser214, 324, and Thr 377 by PKC; and Ser416. Where the Ser-Pro or Thr-Pro sites have been found mostly in the flanking regions are affected by several proline-directed kinases, such as MAP [94]. These phosphorylated kinases specified regions are the best powerful tools for the diagnostic markers and demonstrate in brain samples of AD and Parkinson’s diseases [95,96].

In postmortem brains from persons with AD, over 30 kinase-specific phosphorylation-specific sites were identified as containing phosphorylated tau [97]. In the glycogen synthase kinase-3 (GSK3), nearly 26 sites on the tau protein were detected with antibody-mediated assays. Many other phosphorylation sites involved other kinases, such as cyclin-dependent kinase, glycogen synthase kinase-3, calmodulin-dependent protein kinase-2, abelson murine leukemia virus tyrosine kinase, epidermal growth factor receptor, insulin receptor tyrosine kinase, protein kinase B, and protein kinase D, all of which were well conserved with Ser/Thr [98]. An increase in tau phosphorylation may result in adverse effects associated with AD, such as dementia. The involvement of kinase activity in tau phosphorylation may help elucidate mechanisms underlying tau aggregation in AD [99].

## Biology of Tau

Tau is composed of 477 amino acids with 16 exons (67]. The basic structure of tau differs from other proteins in that tau has about 120 amino acids and 40 amino acid residues in the N-terminal of tau protein. There is an important tau aggregation region in middle with nearly 150 to 240 with prospective combinational residues eight SP or ST. there is a region called proline-rich region, where many kinases were interacted many biological proteins and useful sites for the detection of dementias in the diseased states tauopathies.

## AD and Tauopathies

Aggregation of the insoluble tau protein in a healthy neuron causes adverse effects in a neurofibrillary of glial fibrillary tangles, leading to a pathological neuron. This pathology may in turn cause neurodegenerative disorders, including temporal dementia and other tauopathies. In general, tau promotes the binding of the microtubule assembly, leading to the regulation of neuronal processes. In the normal adult brain, contain 2 to 3 moles of phosphate in the tau protein, in contrast to 3-4-fold (~ 8-mol phosphate/mol protein) in the adult brain affected by AD [100]. Hyperphosphorylated tau protein is considered the primary event leading to diseases with a dissociated filament. The mechanism underlying tau aggregation in dementias is poorly understand and involving kinase and phosphatases in parallel such as phosphorylation, acetylation, ubiquitination, and SUMOylation regulate Tau function and degradation, via temporal and regional regulation of the protein affinity for microtubules disease-associated tau conformations in AD and tauopathies.

The normal phosphorylation of tau forms thread-like filaments around microtubules, resulting in the formation of the stable cytoskeletons. However, excessive phosphorylation results in dissociation of thread-like filaments leading to breakage of tubulin in neuronal damage. Other factors like irregular phosphatases and kinases activity within the brains leads to PHF development leads to death of neuronal cells.

**Figure 3.**
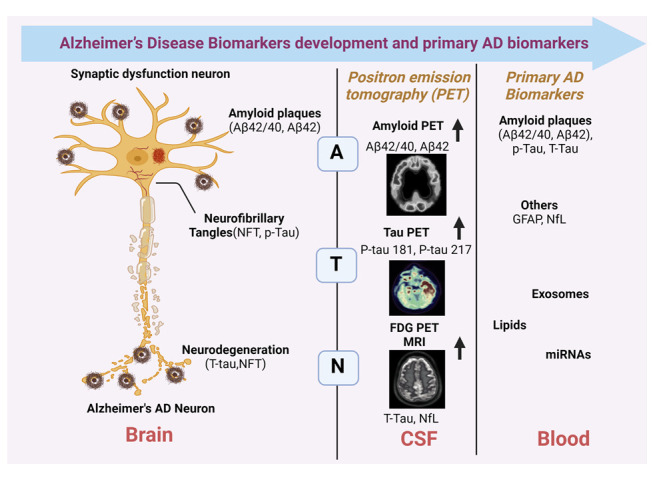
**Alzheimer's biomarkers development in brain, blood and CSF with neuronal degeneration brain imaging technologies to detect amyloid accumulation in the brain using various PET ligands bound to amyloid plaque have emerged**. There are several molecular changes from neurons associated with Aβ=amyloid β, Tau fibrils p-Tau 181, p-Tau 217 in Brain and CSF and other primary biomarkers included with Aβ=amyloid β, Tau fibrils, exosomes, lipids and miRNAs in blood based biomarkers.

## Biomarkers for Tau Pathology

In AD, more tau proteins are released into the extracellular space, which was observed in the CSF of AD patients as increased concentrations of both T-tau, measured using antibodies against mid-domain tau epitopes that are not phosphorylated, and P-tau that is measured using antibody combinations that specifically recognize mid-domain P-tau epitopes (Olsson et al., 2016). These changes in biomarker correlate with each other; however, in AD this association is most pronounced and less pronounced for isolated increases in T-tau [101]. With AD-type neurodegeneration and tangle pathology in autopsy and biopsy studies, there is correlation in CSF T-tau and P-tau [102,103], but not all studies report correlations [104]. This appears to be different from correlation studies between tau PET imaging and CSF tau biomarkers, suggesting differences in how these biomarker modalities reflect the underlying processes in AD.

The severity of acute injury and/or on-going neurodegeneration is reflected by CSF T-tau [105]. Early in the disease process, CSF T-tau is likely increased since increased levels are seen in Aβ-positive cognitively unimpaired individuals (CU). However, the increase in CSF T-tau is not specific to AD, since CSF T-tau levels are the highest in conditions with the most severe neurodegeneration, including Creutzfeldt-Jakob disease, which has been shown to be >10-times higher in T-tau concentrations than dementia [106]. A rapid spike in CSF T-tau is observed following acute brain injury and maintained for weeks before slowly declining to normal levels [107,108]. Higher T-tau values predict a more rapid cognitive decline in AD [109,110] and relate to more rapid hippocampal atrophy and reductions in FDG-PET binding [111, 112], which supports the idea that T-tau levels are related to the intensity of neurodegeneration (Fig. 3).

Several validated commercially available immunoassays targeting threonine 181 (P-tau181) consistently demonstrate an increase in AD. However, other mid-domain P-tau residues (threonine 231, serine 199 and 231) and C-terminal residues (Serine 396 and 404) are also increased in AD [113, 114]. A limited number of studies seem to demonstrate co-linearity but specificity in p-tau 181, 199 and 231 to distinguish AD from other neurodegenerative disorders and aged healthy controls.

## Amyloid and P-Tau Blood-based Markers in AD

In blood-based marker plasma Aβ42/40 ratio, p-tau, serum neurofilament light chain (NfL) are the most advanced biomarkers developed for the diagnosis of AD to monitor the disease-modifying effects. In patients with early AD, there are many assays to measure Aβ in blood and to detect positive amyloid status. Plasma Aβ42/Aβ40 ratio can detect abnormal amyloid status in cognitive impaired patients. In the same way, to Aβ isoforms, several peptides form of p-tau such as p-tau181, p-tau217 and p-tau231 can be detected in AD plasma samples. In AD, plasma p-tau217 correlates with tau tangles in AD, but not in non-AD pathologies, which signifies that p-tau toxicity can be used as biomarkers of AD [115]. However, P-tau217 levels appear to be elevated in the presence of Aβ plaques but not in the absence of Aβ plaques as occurs in primary age related tauopathies or other non-AD tauopathies.

## Combination of Tau and Aβ as Biomarkers

As noted in this review in detail, AD is characterized by both Aβ (A) deposits as Aβ plaques and tau (T) protein deposits as NFT. Due to this main pathological characterization, both Aβ and tau may be used as biomarkers for therapeutic intervention of AD. This is considered as A+T+ AD, but by using CSF biomarkers some studies have observed A-T+ in CSF of AD patients, indicating that some AD patients have only abnormal levels of Aβ and normal level of tau [116, 117].

**Figure 4.**
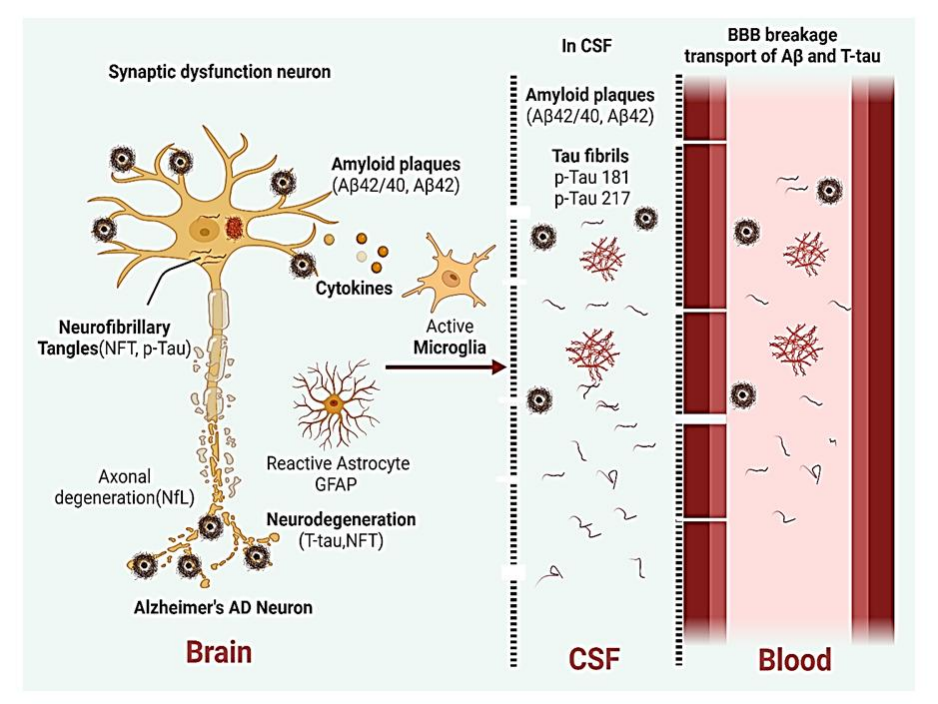
**Alzheimer's disease has a complex pathophysiology**. Fluid-based biomarkers that can be reliably measured in brain, blood and CSF: Aβ, p-Tau, NfL, and GFAP. Biomarkers with strong potential in CSF only include Aβ=amyloid β, Tau fibrils p-Tau 181, p-Tau 217. BBB leakage at some regions transportation of these Aβ, Tau fibrils can be detected in blood used for biomarkers in AD.

In terms of establishing tau and Aβ as biomarkers, the lack of ultrasensitive techniques has impeded development of AD biomarkers in the past. Now there are various ultrasensitive technologies such as SIMOA and IMR technologies that make the development of AD blood-based biomarkers promising and thus will allow us to further understand AD [118]. Furthermore, other conditions that have similar pathologies to AD such as Lewy body dementia are currently being studied using combination of tau and Aβ biomarkers using CSF [119]. Various other fluids besides blood and CSF such as oral, ocular, and olfactory fluids are also being used to obtain biomarkers in order to study various disorders including AD, but none of these fluid biomarkers have been established yet in AD [120] (Fig. 4).

## Biomarker Detection Methods in Recent Years

Recent years have seen tremendous advancements in biomarker detection technologies, especially when it comes to the diagnosis of Alzheimer's disease (AD). Because non-invasive approaches have the potential to transform early identification, intervention, and progression tracking of AD, they are essential in this field [121]. The influence of these important technologies on early AD diagnosis is seen below.

## Neuroimaging Techniques

MRI (Magnetic Resonance Imaging): Advanced MRI methods, including diffusion tensor imaging (DTI) and volumetric MRI, make it possible to see anatomical changes linked to AD in the brain, such as white matter abnormalities and hippocampus atrophy [122]. PET (Positron Emission Tomography): Even in the preclinical phases of Alzheimer's disease, pathological markers of the disease can be seen in the brain by using PET imaging using radiotracers that target tau and amyloid-beta protein clumps [123].

## Cerebrospinal Fluid (CSF) Biomarkers

The examination of CSF biomarkers, such as tau protein, phosphorylated tau, and amyloid beta, offers important insights into the pathogenic mechanisms that underlie AD [124]. Despite requiring an intrusive procedure (lumbar puncture), CSF collection is still one of the most effective ways to identify AD early on.

## Blood-Based Biomarkers

Blood-based biomarkers hold out the possibility of a less invasive and more affordable method of diagnosing AD [125]. Novel developments in proteomic, metabolomic, and lipidomic profiling have revealed blood biomarkers, including tau protein, neurofilament light chain (NfL), amyloid-beta peptides, and inflammatory markers, that may be linked to AD pathogenesis.

## Digital Biomarkers

Digital technologies, such as wearables and smartphone apps, provide a way to track minute changes in behavior, everyday activities, and cognitive function. These changes may be used as early markers of cognitive decline and to enable remote tracking of the course of disease process in AD. Non-invasive biomarker detection methods have a significant potential impact on the early diagnosis of AD [126]. These technologies can help with timely intervention and individualized treatment plans by enabling earlier diagnosis of AD pathology, which will ultimately improve patient outcomes and quality of life. Furthermore, patients can be included in clinical trials during the preclinical or prodromal stages, when therapies may be most helpful in avoiding or slowing the progression of the disease, thanks to early detection. Furthermore, these technologies have the potential to guide therapeutic decision-making, support the development of novel AD medicines, and track treatment response and disease progression over an extended period of time. To fully achieve the potential of non-invasive biomarker identification in early AD diagnosis, however, more validation, standardization, and integration into clinical practice are necessary.

## Biomarkers Efficacy, Sensitivity, and Specificity in Diagnosing AD

The diagnosis of Alzheimer's disease (AD) usually combines neuroimaging, biomarker analysis, clinical assessment, and cognitive testing. In order to diagnose and track AD at various stages, biomarkers are essential. The effectiveness, sensitivity, and specificity of biomarkers frequently employed in the diagnosis of AD at different stages are summarized below.

## Beta-amyloid (Aβ) and Tau Proteins

*Efficacy*: The proteins Tau and Aβ are important indicators linked to the pathophysiology of AD. AD is indicated by aberrant buildup seen on positron emission tomography (PET) scans and elevated levels of Tau and Aβ in the cerebrospinal fluid (CSF).

*Sensitivity and Specificity:* Aβ42, total Tau, and phosphorylated Tau are CSF biomarkers that are very sensitive and specific in differentiating between AD and other neurodegenerative illnesses as well as healthy individuals [127]. However, according on the disease's stage, their sensitivity and specificity may change. Early on, there may be a drop in Aβ42 levels and an increase in Tau levels, which results in excellent sensitivity and specificity. Nevertheless, specificity may be decreased in later stages if Tau levels rise in other neurodegenerative illnesses.

## Neuroimaging Biomarkers

*Efficacy:* Hippocampal atrophy and hypometabolism in particular brain regions are examples of the structural and functional alterations in the brain linked to AD that can be found using neuroimaging techniques like magnetic resonance imaging (MRI) and PET scans [128].

*Sensitivity and Specificity:* MRI-based measures of hippocampal volume and cortical thickness have demonstrated high sensitivity and specificity, especially in the early stages of AD. PET imaging with tracers targeting Aβ and Tau aggregates can also provide valuable information, although specificity may be lower in later stages due to the presence of these aggregates in other neurodegenerative diseases [129].

## Blood-based Biomarkers

*Efficacy:* Blood-based biomarkers offer a less invasive and more accessible alternative to CSF and neuroimaging biomarkers. Several blood-based biomarkers, including plasma Aβ and Tau levels, neurofilament light chain (NfL), and inflammatory markers, are being investigated for their utility in AD diagnosis [130].

*Sensitivity and Specificity:* Blood-based biomarkers are promising but still undergoing validation. Sensitivity and specificity vary among different biomarkers and assay methods. While some blood-based biomarkers may exhibit good sensitivity, their specificity may be lower due to overlap with other conditions or limitations in detecting early-stage pathology.

## Genetic Biomarkers

*Efficacy:* Genetic factors, particularly mutations in genes such as APP, PSEN1, and PSEN2, can contribute to the risk of developing early-onset familial AD [131]. Genetic testing for these mutations can aid in diagnosing familial AD cases.

*Sensitivity and Specificity:* When identifying those at risk of familial AD, genetic testing for mutations related to the disease offers a high sensitivity and specificity. Only a small percentage of AD patients are caused by these mutations, and their significance for sporadic late-onset AD is restricted. To summarize, the diagnostic performance of biomarkers for Alzheimer's disease (AD) varies according on the type of biomarker and the stage of the disease. For a reliable diagnosis and to track the progression of AD, a multimodal strategy that incorporates clinical examination, cognitive testing, neuroimaging, and biomarker analysis is frequently advised. To enhance early detection and individualized treatment of AD, ongoing research is still being done to improve and validate biomarkers.

## Longitudinal Studies of Alzheimer's Disease Biomarkers

Longitudinal studies of Alzheimer's disease (AD) biomarkers are crucial for understanding the progression of the disease, identifying potential therapeutic targets, and developing effective diagnostic and treatment strategies [132-140]. These studies typically involve tracking individuals over an extended period, often years, to observe changes in various biomarkers associated with AD. Biomarkers can include biological indicators such as levels of certain proteins in the blood or cerebrospinal fluid, neuroimaging findings, and cognitive assessments. Here are some key findings and methodologies from longitudinal studies of AD biomarkers (Table 1).

*Amyloid Beta (Aβ) Accumulation*: Longitudinal studies have shown that Aβ deposition, particularly in the form of amyloid plaques, increases over time in individuals who develop AD. Positron emission tomography (PET) imaging using tracers such as Pittsburgh compound B (PiB) allows for the visualization and quantification of Aβ in the brain [141].

*Tau Pathology*: Tau protein aggregation is another hallmark of AD. Longitudinal studies have demonstrated that elevated levels of tau in cerebrospinal fluid (CSF) or abnormal tau PET imaging correlate with cognitive decline and progression to AD dementia.

*Neuroimaging Changes*: Magnetic resonance imaging (MRI) and PET imaging can reveal structural and functional changes in the brain associated with AD progression. Longitudinal studies have shown patterns of brain atrophy, particularly in regions such as the hippocampus and entorhinal cortex, which are involved in memory and learning.

**Table 1:** A list of long-term biomarker studies that were found through a thorough literature review.

	Study	Biomarkers	Number of measures	Length of the Study	Location	References
**1.**	ADAPET (Alzheimer’s Disease and Positron Emission Tomography)	MRI	50	5 y	South Korea	Kim et al., 2015[132]; Cho et al., 2014[133].
**2.**	AIBL (The Australian Imaging, Biomarker &Lifestyle Flagship Study of Ageing)	MRI, PiB-PET, blood	1100	4.5 y	Australia	Burnham et al., 2014[134]; Pietrzak et al., 2015[135].
**3.**	BIOCARD	CSF, MRI, Plasma	349	17 y	USA	Miller et al., 2015[136]; Younes et al., 2014[137].
**4.**	Göteborg MCI study	CSF, MRI, SPECT, EEG, Plasma	226	10 y	Sweden	Bjerke et al., 2009[138]; Eckerstrom et al., 2015[139].
**5.**	DIAN (Dominantly Inherited Alzheimer Network)	CSF, PiB, FDG, PET, MRI	122	1.1-2.1 y	USA, Australia, Europe, Asia, and South America	Benzinger et al., 2013[140].

*Cognitive Decline*: Longitudinal assessments of cognitive function, including memory, executive function, and processing speed, are integral to tracking AD progression. These studies often utilize standardized neuropsychological tests administered at multiple time points to monitor changes in cognitive abilities.

*Genetic Risk Factors*: Longitudinal studies in individuals with genetic predispositions to AD, such as those carrying mutations in genes like APP, PSEN1, and PSEN2, provide insights into the earliest stages of disease development and progression [142].

*Biomarker Trajectories:* Longitudinal analyses of biomarker data allow researchers to characterize individual trajectories of change over time and identify distinct patterns associated with AD progression. Understanding these trajectories is essential for predicting disease onset and monitoring treatment responses.

*Clinical Trials*: Longitudinal studies play a crucial role in evaluating the efficacy of potential AD treatments by tracking biomarker changes in response to interventions. These trials often incorporate biomarker endpoints, such as reduction in Aβ or tau levels, as surrogate markers of treatment effectiveness.

Overall, longitudinal studies of AD biomarkers offer valuable insights into the natural history of the disease, facilitate early detection, and inform the development of targeted interventions aimed at slowing or preventing AD progression. However, challenges such as participant attrition, variability in biomarker measurement techniques, and the need for long-term follow-up remain important considerations in designing and interpreting these studies [132-140].

## Emerging Therapeutic Interventions Based on AD Biomarkers

Alzheimer's disease (AD) biomarkers play a crucial role in the early detection and monitoring of the disease progression. As research in this area continues to evolve, emerging therapeutic interventions are being developed based on these biomarkers. Here are some notable examples:

*Tau-targeted Therapies:* Abnormal accumulation of tau protein is a hallmark of AD. Therapies targeting tau pathology are being explored. Some approaches include monoclonal antibodies that aim to clear tau aggregates from the brain or inhibit the spread of pathological tau.

*Beta-amyloid Clearance Therapies:* Beta-amyloid plaques are another key pathological feature of AD. Various drugs are being developed to target beta-amyloid, including monoclonal antibodies designed to clear beta-amyloid from the brain or inhibit its production.

*Inflammation Modulation:* Neuroinflammation is increasingly recognized as a contributing factor to AD progression. Therapies aimed at modulating neuroinflammatory responses are being investigated, including drugs targeting microglia activation and cytokine signaling.

*Neuroprotective Agents:* Some therapeutic strategies focus on protecting neurons from degeneration and promoting neuronal health. This includes drugs that target oxidative stress, mitochondrial dysfunction, and synaptic loss.

*Precision Medicine Approaches:* With advancements in biomarker identification, there's a growing interest in precision medicine approaches for AD treatment. These approaches aim to tailor therapies based on individual patient characteristics, including genetic risk factors, biomarker profiles, and disease stage.

*Combination Therapies:* Given the complexity of AD pathology, combination therapies targeting multiple aspects of the disease are being explored. These may involve combining drugs that target beta-amyloid, tau, neuroinflammation, and other pathological processes to achieve synergistic effects.

*Lifestyle Interventions*: Emerging evidence suggests that lifestyle factors such as diet, exercise, cognitive stimulation, and sleep quality may influence AD risk and progression. Therapeutic interventions focusing on lifestyle modifications are being investigated as adjunctive or preventive strategies for AD [143]. *Gene Therapy:* Gene therapy approaches aimed at modifying or replacing dysfunctional genes implicated in AD pathology are under development. These may involve gene editing techniques such as CRISPR-Cas9 or gene delivery methods to introduce therapeutic genes into the brain [144].

*Exosome-based Therapies:* Exosomes, small vesicles secreted by cells, are being explored as potential therapeutic vehicles for delivering drugs or biomolecules to target cells in the brain. Exosome-based therapies hold promise for targeted delivery of therapeutic agents to specific brain regions affected by AD pathology.

*Blood-based Biomarkers and Therapies:* Research into blood-based biomarkers for AD detection and monitoring is advancing rapidly. Therapeutic interventions targeting these blood-based biomarkers, such as circulating beta-amyloid or tau, are being investigated for their potential to diagnose and treat AD at early stages. Overall, the development of therapeutic interventions based on AD biomarkers represents a promising approach to tackling this devastating disease. However, further research and clinical trials are needed to validate the safety and efficacy of these emerging treatments.

*Lifestyle Interventions:* Lifestyle factors such as diet, exercise, cognitive stimulation, social engagement, and management of cardiovascular risk factors have been shown to influence the risk of developing AD and may affect disease progression. Adherence to a Mediterranean diet, rich in fruits, vegetables, whole grains, fish, and olive oil, has been associated with a lower risk of AD [145]. Regular physical exercise has been shown to have neuroprotective effects and may help reduce the risk of cognitive decline in older adults. Cognitive training programs and social engagement activities have demonstrated some benefits in preserving cognitive function and delaying the onset of AD symptoms [146].

## New Biomarker Discovery

Biomarkers are measurable indicators of biological processes or disease states. In the context of AD, biomarkers play a crucial role in early diagnosis, monitoring disease progression, and assessing the efficacy of potential treatments. Traditional biomarkers for AD include levels of amyloid-beta and tau proteins in cerebrospinal fluid (CSF), as well as amyloid and tau imaging using positron emission tomography (PET) scans. Recent research efforts have focused on discovering novel biomarkers that can improve the accuracy of early diagnosis and provide insights into the underlying pathophysiological processes of AD. These emerging biomarkers include neuroinflammatory markers, synaptic proteins, blood-based biomarkers, and advanced neuroimaging techniques aimed at detecting subtle changes in brain structure and function.

In summary, lifestyle interventions, and the discovery of new biomarkers all contribute to our understanding of Alzheimer's disease and offer avenues for early detection, prevention, and targeted therapeutic interventions. Ongoing research in these areas is essential for advancing our ability to diagnose, treat, and ultimately prevent AD.

## Caregivers’ Experiences with AD Patients and Management

Caregivers play a vital role in supporting individuals diagnosed with Alzheimer's disease (AD) throughout their journey. Understanding their experiences with diagnosis and management provides valuable insights into the challenges they face and the support they need. Here are some common themes based on caregivers' experiences:

*Emotional Impact of Diagnosis:* Caregivers often experience a range of emotions when their loved ones are diagnosed with AD, including shock, denial, sadness, and anxiety about the future [147]. They may struggle to come to terms with the reality of the diagnosis and the prospect of providing care for someone with a progressive neurodegenerative disease.

*Navigating the Diagnostic Process*: Caregivers may encounter challenges in navigating the diagnostic process, including delays in receiving a diagnosis, difficulty accessing appropriate healthcare services, and uncertainty about the accuracy of the diagnosis. Clear communication and support from healthcare professionals are essential during this time to help caregivers understand the implications of the diagnosis and access available resources.

*Managing Symptoms and Care Needs:* Caregivers are often responsible for managing the day-to-day symptoms and care needs of individuals with AD. This can include assisting with activities of daily living, managing medication regimens, coordinating medical appointments, and addressing behavioral and psychological symptoms such as agitation, aggression, and wandering.

*Impact on Daily Life:* Providing care for someone with AD can have a significant impact on caregivers' daily lives, including disruptions to work, social activities, and personal well-being [148]. Many caregivers experience increased stress, fatigue, and social isolation as they navigate the demands of caregiving while balancing their own needs and responsibilities.

*Financial and Legal Considerations:* Caregivers may face financial strain due to the costs associated with caregiving, including medical expenses, home modifications, and loss of income from reduced work hours or employment opportunities. Navigating complex legal and financial issues, such as advance care planning, power of attorney, and long-term care options, can add to the burden of caregiving.

*Seeking Support and Resources:* Caregivers benefit from access to support services and resources to help them cope with the challenges of caregiving and maintain their own health and well-being. This may include support groups, respite care, counseling services, educational materials, and assistance from community organizations and healthcare professionals.

Overall, caregivers' experiences with AD diagnosis and management underscore the importance of comprehensive support systems that address their emotional, practical, and informational needs. By understanding and addressing the challenges faced by caregivers, we can better support them in their crucial role of caring for individuals with AD while preserving their own health and well-being.

## AD Management and Available Diagnostic Tools

Alzheimer's disease (AD) management encompasses a multifaceted approach that includes early screening programs and access to diagnostic tools. Here's an overview of key components. An early screening program aims to identify individuals at risk for AD or in the early stages of the disease before significant cognitive impairment occurs.

These programs may target specific populations, such as older adults, individuals with a family history of AD, or those with known risk factors such as cardiovascular disease or genetic predisposition. Screening methods may include cognitive assessments, biomarker testing, genetic testing, and neuroimaging techniques to detect early signs of cognitive decline or underlying pathological changes associated with AD [149]. Early screening allows for timely intervention and access to support services, clinical trials, and potential treatments aimed at slowing disease progression or improving quality of life.

## Access to Diagnostic Tools

Access to accurate diagnostic tools is essential for confirming a diagnosis of AD and ruling out other potential causes of cognitive impairment. Diagnostic tools for AD include comprehensive clinical evaluations, cognitive assessments, laboratory tests, neuroimaging (such as MRI, PET scans), and biomarker testing (such as cerebrospinal fluid analysis for amyloid-beta and tau proteins) [150]. Biomarker testing, in particular, is becoming increasingly important for early and accurate diagnosis of AD, as it can detect pathological changes in the brain associated with the disease before symptoms manifest. Improving access to diagnostic tools requires efforts to increase awareness among healthcare providers, enhance training in dementia diagnosis and management, reduce barriers to testing (such as cost or availability), and promote collaboration between primary care physicians, specialists, and diagnostic centers.

## Comprehensive Management Approach

AD management involves a comprehensive approach that addresses not only cognitive symptoms but also behavioral, psychological, and functional aspects of the disease. Treatment strategies may include pharmacological interventions (such as cholinesterase inhibitors and memantine) to manage cognitive symptoms and behavioral disturbances. Non-pharmacological interventions, such as cognitive stimulation therapy, physical exercise, dietary modifications, and caregiver support programs, are also important components of AD management. Individualized care plans should be tailored to the specific needs and preferences of each patient and their caregivers, with regular monitoring and adjustments as the disease progresses.

## Comorbidity Profile of AD Patients

AD is associated with comorbid disorders. Data from clinical and genetic research suggest that chronic conditions like diabetes, depression, heart disease, and inflammatory bowel disease may make certain individuals more vulnerable to AD. Comorbid illnesses can arise before or at the same time as AD and can affect the overall clinical status and progression of the illness. Linkages between AD and other chronic diseases have been demonstrated by multiple lines of evidence, including atherosclerosis [151] diabetes (152), stroke [153], cardiovascular disease [154,155], depression [156], and inflammatory bowel disease [157]; A third of AD cases in the US were shown to be associated with seven modifiable risk factors: lack of exercise, smoking, diabetes, obesity, hypertension, and low levels of education [158]. Reducing all seven risk variables by 10-25% may be able to stop up to 3 million AD cases globally and up to approx. 0.5 million cases in the United States [159]. Systems-based biology studies have revealed common genetic factors and dysregulated pathways that could explain the connection between comorbid disorders in AD. The precise processes behind the emergence of medical comorbidities in AD are yet unknown. Novel approaches to therapeutic interventions may arise from an understanding of the molecular mechanisms underlying comorbid disorders in AD. In the last part of the review, we’ll go over the most common comorbidities in AD, along with how they affect AD patients' clinical care.

## AD, Diabetes, and Glucose Metabolism

Patients with diabetes have a high risk of developing cognitive decline or at least diabetes accelerates the earlier onset of cognitive decline. For cognitive impairment or dementia, especially those closely associated with AD, diabetes mellitus is regarded as an independent risk factor [160,161]. Some academics have referred to AD as type 3 diabetes mellitus because of the higher likelihood of late-onset AD in diabetic patients [162]. One cohort study revealed that diabetes in midlife was associated with a 19% greater cognitive decline in 20 years [163]. While a cross sectional study linked diabetes to cognitive decline [164], another 10 years follow up cohort study found Diabetes and HbAc1 levels associated with cognitive decline and prediabetes associated with an increased risk of dementia [165]. One systematic review suggested that the incidence of dementia was higher in diabetes compared to non-diabetic patients [166]. Marseglia et al. [167] confirmed diabetes and prediabetes accelerated cognitive impairment [167] and this is supported by a meta-analysis linking Diabetes with a 1.25-1.9-fold increase in cognitive impairment and dementia [168]. In another article Fontbonne et al. [168] showed diabetes associated with a 2-3-fold increase risk of dementia [169]. A cohort study showed diabetes or impaired fasting glucose was present in 81% of AD patients [170]. Diabetic patients may be at a higher risk of acquiring AD if they have dysfunction in insulin signaling, as this can lead to tau hyperphosphorylation through GSK-3β. [171,172] The likelihood of diabetic patients developing Alzheimer's disease was also explored by assessing the presence of AD biomarkers in their CSF and plasma. The plasma levels of Aβ1-42 and t-tau were considerably higher in the diabetes patients than healthy adult controls indicating the high risk for AD in diabetic patients with regards to plasma biomarkers [173]. Though some publications reported no relationship between HbA1c or blood glucose level and CSF Aβ1-42, pTau level [174,175], diabetic condition promoted higher level of CSF p-tau in AD patients carrying the APOE4 genotype [176]. In APOE4 carriers, insulin resistance also upregulated CSF p-tau levels in AD [177].

Recent studies have confirmed that insulin resistance (IR) is a risk factor for AD. Under typical circumstances, elevated levels of plasma glucose prompt the production of insulin by pancreatic β-cells, therefore lowering glucose levels. However, in insulin resistance, cells become less sensitive to insulin, which raises blood sugar levels. Lately it was reported that Impaired insulin sensitivity is linked to adverse changes in plasma level of AD-related biomarkers (neurofilament light chain, glial fibrillary acidic protein, Aβ 42/40, and phosphorylated tau (p-tau) 181 and 231). Insulin resistance not only induces tau hyperphosphorylation through GSK3β [178] but also upregulates the production of Aβ-protein via activation of the MAPK pathway by pro-inflammatory cytokines like tumor necrosis factor-α, interleukin-1β, and interferon-γ stimulate γ-secretase [179].

## AD and Cardiovascular Disease

It has long been perceived that cardiovascular risk factors are intimately linked to the onset of AD. An increasing number of older people have hypertension as a risk factor for Alzheimer's. Petrovitch et al. [180] showed that midlife high blood pressure linked to the formation of neurofibrillary tangles and neurotic plaques in AD [180]. Blood pressure variability is also linked to AD pathophysiology [181]. In a cross-sectional study in older individuals, elevated blood pressure was associated with lower levels of plasma Aβ1-42 and higher levels of total tau and Ptau181:Aβ1-42 ratio suggesting higher blood pressure variability is linked to plasma biomarkers of increased AD risk [182]. Increased CSF phosphorylated tau, increased CSF total tau, and decreased CSF β-amyloid levels were associated with visit-to-to-visit blood pressure variability indicating its role in altering AD biomarkers [183].

Which can be controlled by taking antihypertensive medication as reported in another occasion as use of antihypertensive drugs lowered incidence of AD and reduced risk for dementia [184,185]. In a similar Fashion Peila et al. [186] also reported in a population-based cohort study that men who use antihypertensive medications are at a lower risk of developing dementia and cognitive impairment [186].

AD has been connected to cardiovascular conditions such as coronary heart disease, atrial fibrillation, and stroke. The initial research identified a possible connection between AD and cerebrovascular complications linked brain infarcts to higher rates of dementia and cognitive loss in comparison to people without brain lesions [187]. Earlier in 1997, Hoffman and colleagues showed that atherosclerosis is one of the major risk factors for AD and dementia [188]. Later in 2010, Bunch et al. association between atrial fibrillation and AD [189]. Recently it was confirmed that stroke increased the risk of AD.

## AD and Depression

There is a link between having a history of depression and a higher chance of AD in later life. Older adults who suffer from depression frequently experience weariness, loss of appetite, sleep difficulties, and low energy, among other symptoms [156]. The primary component of AD plaques, Aβ-42, interfere with key neurotransmitter systems that are connected to depression [190]. An investigation using population-based case-control data revealed a strong positive correlation between AD and episodic depression [191]. According to a different prospective longitudinal investigation, having a low depressive mood somewhat raised the chance of AD [192], which is further confirmed by another systemic review[156]. Hence, scientists suggested that old age depression might be a predictor of subsequent dementia [193]. CSF level of Aβ has also been studied in conjunction with major depression in elderly individuals. CSF Aβ-42 levels were significantly lower in the cognitively intact elderly individuals with major depression relative to the comparison group [194]. Gudmundsson et al. [195] also reported that the elderly women with depression had higher CSF levels of Aβ-42 than comparison subjects. A pilot study suggested that increased plasma Aβ42 and Aβ42/40 ratios are present in geriatric depression [196] while others reported that the elderly with depression had lower plasma Aβ42 than those without depression [197]. Moreover, Nascimento et al., [198] suggested that individuals with depression in their later years exhibit notable variations in their Aβ metabolism, which may indicate that they are more susceptible to AD [198]. Future studies should be done to confirm these findings and to determine their relationship to cognitive decline and CSF and plasma Aβ42 taking other comorbidities into account.

## AD, Obesity, and Lipid Profile

A sedentary lifestyle, excessive consumption of saturated fat, or both bring on a major global health concern, obesity, which is defined by as Body Mass Index (BMI) being ≥30 kg/m^2^. Obesity is considered as a significant risk factor of AD [199, 200]. Adipose tissue secretes the adipokine leptin, which rises with obesity and is thought to be the mediator of inflammation that leads to dementia [201]. Several prospective studies suggested midlife obesity as a risk factor for dementia. A longitudinal analysis was conducted on 6,583 members; where 15.9% of participants were diagnosed with dementia and a threefold increased risk of dementia were reported in case of individuals having higher abdominal diameter in their midlife [202]. In the Baltimore Longitudinal Study of Aging (BLSA), they studied influence of midlife adiposity on age at onset (AAO) of AD and found AD is predicted to begin 6.7 months earlier for every unit increase in midlife BMI [203]. A review on analyzing data from eight different longitudinal population-based studies found a significantly increased risk of dementia with elevated BMI [204]. Obesity was also identified as a substantial risk factor for the development of AD and all-cause dementia in a number of other meta-analyses and systematic reviews [205]. Research reveals that dyshomeostasis of plasma Aβ, specifically a low 42/40 ratio, is associated with an increased risk of AD. The plasma Aβ42/40 ratio was seen to be 17.54% lower in individuals with an obese BMI than in individuals with a lean BMI [206]. The strong correlation between plasma levels of Aβ42 and BMI has been found in healthy patients in another study [207].

Dyslipidemia has been successively incriminated in the development of AD [208]. Dyslipidemia can be a result of genetic and diet factors where plasma level of lipoproteins, fatty acids (saturated or unsaturated), triglycerides (TG), total cholesterol (TC), and phospholipids are altered. Studies are ongoing to see if they have the potential to be regarded as a biomarker for dementia, even though not all observational studies have found elevated serum cholesterol levels among those who acquire AD pathology [209].

According to observational research, having high cholesterol in middle age is an independent risk factor for the progression of AD at a later stage [210]. In one large retrospective cohort study, risk for Ad increased by 57% for having high total cholesterol in early life [211]. Total cholesterol was also greater in AD patients than in elderly controls in a trial conducted in a clinical setting; but there was no significant difference between the two groups' triglyceride levels [212]. However, from the result of brain autopsy, Aβ plaques were reported for the individuals having early life elevated low-density lipoprotein cholesterol (LDL-C) and TC [213-214]. In line to this, another article found a significant increase in Aβ and tau deposition in higher LDL-C group than in lower LDL-C group [215].

**Figure 5.**
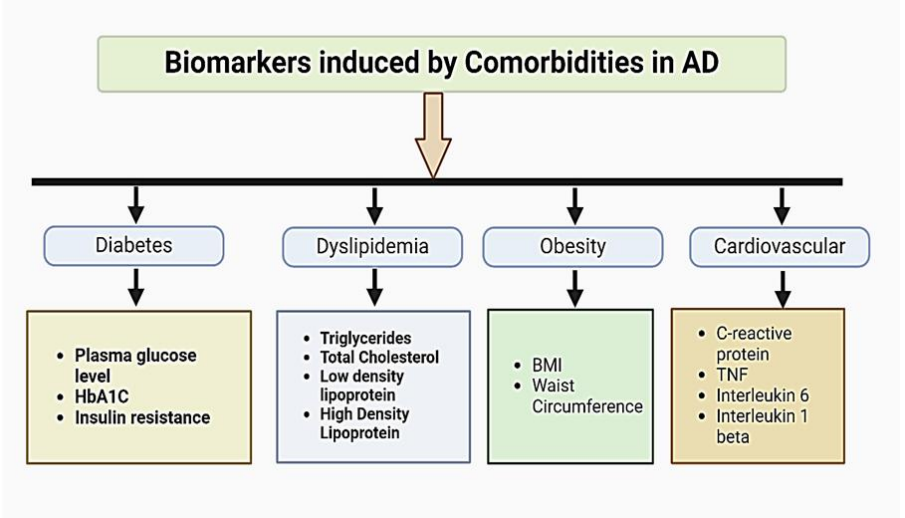
**AD patients have several comorbidities like hypertension, atherosclerosis, diabetes, dyslipidemia, and obesity**. Comorbidity status marked by several biomarkers also accelerates AD progression. We should consider these biomarkers while assessing AD patients with comorbidities.

The concomitant relationship between fasting plasma TC, TGs, and lipid levels and AD has also been extensively studied in another epidemiologic research [216,217]. Changes in biochemical parameters like lipid profile may be present long before the symptomatic onset of AD and might be indicative to the disease progression. Based on these epidemiologic and observational data, it could be inferred that development of the pathogenesis of AD might be instigated by dyslipidemia. Most intriguing part is Apolipoprotein E (ApoE) is the major cholesterol transporter in the brain, and increased expression of this gene, especially in ApoE4/E4, is associated with increased risk of late onset familial and sporadic forms of AD [218]. Furthermore, it has been demonstrated *in vitro* that lower cholesterol levels elevate α-secretase activity and shift the equilibrium away from the nonsoluble Aβ product [219]. Considering all these clinical, molecular and cellular evidence, cholesterol level evolved as a major biomarker in the investigation of AD and has emerged as a target cellular event for developing potential therapeutic treatments (Fig. 5).

## Conclusions and Future Directions

The forerunner has generated a large and considerable method and procedure and conducted a great deal of research on CSF AD biomarkers. Furthermore, it is suggested that innovative biomarkers be used as noninvasive indicators in AD research going advancing. With the emergence of blood-based biomarkers, we have never-before-seen opportunities to better diagnose patients, track their progress, and tailor their care. Though the exact process underlying the start of AD is unknown, it will be extremely difficult to identify the biomarkers that best capture the transition from non-dementia to dementia in future research in early detection. However, in combination with other risk factors, will provide a novel solution that may revolutionize the early diagnosis of AD.
